# Remedies and Their Abbreviations

**Published:** 1889

**Authors:** Edmund J. Lee


### REMEDIES AND THEIR ABBREVIATIONS.

Abies-c., Abies Canadensis.
Abies-n., Abies nigra.
Abrot., Abrotanum.
Absin, Absinthium.
Acal., Acalypha Indica.
Acet-ac., Acetic acid.
Acon., Aconitum napellus.
Acon-a., Aconitum anthora.
Acon-c., Aconitum cammarum.
Acon-f., Aconitum ferox.
Acon-l., Aconitum lycoctonum.
Acon-s., Aconitum septentrionale.
Actæa spicata. See Cimicifuga.
Æs-g., Æsculus glabra.
Æsc-h., Æsculus hippocastanum.
Æth., Æthusa.
Agar-em., Agaricus emeticus.
Agar, Agaricus muscarius.
Agar-ph., Agaricus phalloides.
Agn., Agnus castus.
Ailan., Ailanthus.
Alco., Alcohol.
Alet., Aletris farinosa.
All-c., Allium cepa.
All-s., Allium sativum.
Aloe, Aloe.
Alumn., Alumen.
Alum., Alumina.
Ambr., Ambra.
Ambro., Ambrosia artemisiæfolia.
Ammc., Ammoniacum.
Am-ac., Ammonium aceticum.
Am-ben., Ammonium benzoicum.
Am-bro., Ammonium bromidum.
Am-c., Ammonium carbonicum.
Am-cau., Ammonium causticum.
Am-i., Ammonium iodatum.
Am-m., Ammonium muriaticum.
Am-n., Ammonium nitricum.
Am-p., Ammonium phosphoricum.
Amph., Amphisbœna.
Amyg., Amygdalæ amaræ aqua.
Aml-n., Amyl nitrite.
Aml-ch., Amylamine chlorohydrate.
Anac., Anacardium.
Anag., Anagallis.
Anan., Anantherum.
Ang., Angustura.
Anil., Anilinum.
Anis., Anisum stellatum.
Anth-n., Anthemis nobilis.
Anthr., Anthrakokali.
Ant-a., Antimonium arsenitum.
Ant-m., Antimonium muriaticum.
Ant-cr., Antimonium crudum.
Ant-ox., Antimonium oxidum.
Ant-s., Antimonium sulf. auratum.
Ant-t., Antimon. et potass. tart.
Apis, Apis.
Ap-g., Apium graveolens.
Aph., Aphis chenopodii glauci.
Apoc., Apocynum cannabinum.
Apom., Apomorphine.
Aq-m., Aqua marina.
Aq-p., Aqua petra.
Aral., Aralia racemosa.
Aran., Aranea.
Aran-d., Aranea diadema.
Aran-s., Aranea sciencia.
Argem., Argemone.
Arg-c., Argentum cyanidum.
Arg., Argentum metallicum.
Arg-m., Argentum muriaticum.
Arg-n., Argentum nitricum.
Arn., Arnica.
Ars., Arsenicum album.
Ars-h., Arsenicum hydrogenisatum.
Ars-i., Arsenicum iodatum.
Ars-m., Arsenicum metallicum.
Ars-s-f., Arsenicum sulphuratum flavum.
Ars-s-r., Arsenicum sulphuratum rubrum.
Artemisia abrotanum. See Abrotanum.
Arum-d., Arum dracontium.
Arum-i., Arum italicum.
Arum-m., Arum maculatum.
Arum-t., Arum triphyllum.
Arund., Arundo mauritanica.
Asaf., Asafœtida.
Asar., Asarum.
Asc-c., Asclepias cornuti (Syriaca).

Asc-t., Asclepias tuberosa.
Asim., Asimina triloba.
Aspar., Asparagus.
Aster., Asterias rubens.
Astr., Astragalus Menziesii.
Ath., Athamanta.
Atro., Atropinum.
Aur., Aurum.
Aur-ful., Aurum fulminans.
Aur-m., Aurum muriaticum.
Aur-s., Aurum sulphuratum.

Bad., Badiaga.
Bals., Balsamun Peruvianum.
Bapt., Baptisia.
Bart., Bartfelder (acid spring).
Bar-ac., Baryta acetica.
Bar., Baryta carbonica.
Bar-m., Baryta muriatica.
Bell., Belladonna.
Bell-p., Bellis perennis.
Benz., Benzinum.
Benz-n., Benzinum nitricum.
Benz-ac., Benzoic acid.
Benzoin, Benzoin.
Berbn., Berberinum.
Berb., Berberis.
Bism., Bismuthum oxidum.
Bol., Boletus laricis.
Bon., Bondonneau.
Borax, Borax.
Bor-ac., Boracium acidum.
Bot., Bothrops lanceolatus.
Bov., Bovista.
Brach., Brachyglottis.
Brass., Brassica napus.
Brom., Bromium.
Bruc., Brucea antidysenterica.
Brucn., Brucinum.
Bry., Bryonia.
Bufo, Bufo.
Buf-s., Bufo sahytiensis.

Cact., Cactus.
Cadm., Cadmium sulphuratum.
Cad-br., Cadmium bromatum.
Cai., Cainca.
Caj., Cajuputum.
Calad., Caladium.
Calc-ac., Calcarea acetica.
Calc-c., Calcarea carbonica.
Calc-cau., Calcarea caustica.
Calc-cl., Calcarea chlorata.
Calc-f., Calcarea fluorata.
Calc-i., Calcarea iodata.
Calc-m., Calcarea muriatica.
Calc-p., Calcarea phosphorica.
Calc-s., Calcarea sulphurica.
Calen., Calendula.
Calo., Calotropis.
Calth., Caltha.
Camph., Camphora.
Canc-f., Cancer fluviatilis.
Canna, Canna.
Cann-i., Cannabis indica.
Cann-s., Cannabis sativa.
Canth., Cantharis.
Caps., Capsicum.
Carb-an., Carbo animalis.
Carb-v., Carbo vegetabilis.
Carb-ac., Carbolic acid.
Carbn., Carboneum.
Carbn-cl., Carboneum chloratum.
Carbn-h., Carboneum hydrogenisatum.
Carbn-o., Carboneum oxygenisatum.
Carbn-s., Carboneum sulphuratum.
Card-b., Carduus benedictus.
Card-m., Carduus marianus.
Carl., Carlsbad.
Casc., Cascarilla.
Cass., Cassada.
Cast-v., Castanea vesca.
Cast-eq., Castor equi.
Castor., Castoreum.
Cata., Catalpa.
Caul., Caulophyllum.
Caus., Causticum.
Cedr., Cedron.
Celt., Celtis.
Cent., Centaurea tagana.
Ceph., Cephalanthus.
Cer-b., Cereus bonplandii.
Cer-s., Cereus serpentinus.
Cerv., Cervus.
Cham., Chamomilla.
Chel., Chelidonium majus.
Chen-a., Chenopodium anthelminticum.
Chen-v., Chenopodium vulvaria.
Chim., Chimaphila.
Chin., China.
Chin-a., Chininum arsenicosum.
Chin-m., Chininum muriaticum.
Chin-s., Chininum sulphuricum.
Chlol., Chloralum.
Chlf., Chloroformum.
Chlo., Chlorum.
Chr-ac., Chromium acidum.
Chr-ox., Chromium oxidatum.
Cic-m., Cicuta masculata.
Cic., Cicuta virosa.
Cimx., Cimex.
Cimic., Cimicifuga.
Cina, Cina.
Cinch., Cinchoninum sulphuricum.

Cinnb., Cinnabaris.
Cinnm., Cinnamomum.
Cist., Cistus.
Cit-ac., Citric acid.
Cit-l., Citrus limonum.
Cit-v., Citrus vulgaris.
Clem., Clematis.
Cob., Cobaltum.
Coca, Coca.
Cocci., Coccionella.
Cocc., Cocculus.
Cocc-c., Coccus cacti.
Coch., Cochlearia.
Cod., Codeinum.
Coff., Coffea cruda.
Coff-t., Coffea tosta.
Coffn., Coffeinum.
Colch., Colchicum.
Coll., Collinsonia.
Coloc., Colocynthis.
Colocn., Colocynthinum.
Colost., Colostrum.
Com., Comocladia.
Conch., Conchiolinum.
Con., Conium.
Conin., Coninum.
Conv-a., Convolvulus arvensis.
Conv-d., Convolvulus duartinus.
Cop., Copaiva.
Coral., Corallium rubrum.
Cori-m., Coriaria myrtifolia.
Cori-r., Coriaria ruscifolia.
Corn., Cornus circinata.
Corn-f., Cornus florida.
Corn-s., Cornus sericea.
Cot., Cotyledon.
Creos., Creosotum.
Croc., Crocus.
Crotal., Crotalus horridus.
Crot-c., Crotalus cascavella.
Croton, Croton tiglium.
Cub., Cubeba.
Cund., Cundurango.
Cupr., Cuprum.
Cupr-ac., Cuprum aceticum.
Cupr-ar., Cuprum arsenicosum.
Cupr-m., Cuprum muriaticum.
Cupr-n., Cuprum nitricum.
Cupr-s., Cuprum sulphuricum.
Cur., Curare.
Cyc., Cyclamen.

Daph., Daphne Indica.
Delph., Delphinus.
Der., Derris pinnata.
Dig., Digitalis.
Dign., Digitalinum.
Dios., Dioscorea.
Dirc., Dirca palustris.
Dol., Dolichos pruriens.
Dor., Doryphora.
Dros., Drosera.
Dub., Duboisia.
Dulc., Dulcamara.

Elaps, Elaps.
Elat., Elaterium.
Elæ., Elæis guineensis.
Emet., Emetinum.
Equ., Equisetum.
Erec., Erechthites.
Erig., Erigeron.
Ery-a., Eryngium aquaticum.
Ery-m., Eryngium maritimum.
Eryth., Erythrophlæum.
Erio., Eriodictyon.
Eth., Ether.
Eth-n., Ethyl nitrate.
Eucal., Eucalyptus.
Eugen., Eugenia jambos.
Euo., Euonymus Europæus.
Eup-per., Eupatorium perfoliatum.
Eup-pur., Eupatorium purpureum.
Euph., Euphorbium.
Euphr., Euphrasia.
Eupi., Eupion.

Fago., Fagopyrum.
Fel., Fel tauri.
Ferr., Ferrum.
Ferr-i., Ferrum iodatum.
Ferr-m., Ferrum muriaticum.
Ferr-p., Ferrum phosphoricum.
Ferr-s., Ferrum sulphuricum.
Fil., Filix mas.
Fluor-ac., Fluoricum acidum.
For., Formica.
Frag., Fragaria.
Fran., Franzensbad.
Frax., Fraxinus.

Gad., Gadus morrhua.
Gal-ac., Gallicum acidum.
Gamb., Gambogia.
Gas., Gastein.
Gels., Gelsemium.
Gen., Genista.
Gent., Gentiana cruciata.
Gent-l., Gentiana lutea.
Ger., Geranium maculatum.
Gett., Gettysburg.
Gins., Ginseng.
Glon., Glonoin.
Gnap., Gnaphalium.

Goss., Gossypium.
Gran., Granatum.
Graph., Graphites.
Grat., Gratiola.
Grin., Grindelia.
Guan., Guano.
Guara., Guarana.
Guare, Guarea.
Guai., Guaiacum.
Gymn., Gymnocladus.

Hæmat., Hæmatoxylon.
Hall, Hall.
Ham., Hamamelis.
Hell., Helleborus niger.
Helon., Helonias.
Hepar, Hepar sulphuris calcareum.
Hipp., Hippomanes.
Hura, Hura Brasiliensis.
Hydrs., Hydrastis.
Hydr-ac., Hydrocyanic acid.
Hydrphb., Hydrophobinum.
Hyos., Hyoscyamus.
Hyosn., Hyoscyaminum.
Hyper., Hypericum.
Hypo., Hypophyllum.

Iber., Iberis.
Ign., Ignatia.
Ill., Illicium.
Indm., Indium metallicum.
Indg., Indigo.
Inu., Inula.
Iod., Iodum.
Iodof., Iodoformum.
Ipec., Ipecacuanha.
Ir-fl., Iris florentina.
Ir-fœ., Iris fœtidissima.
Iris, Iris versicolor.

Jabor., Jaborandi.
Jac., Jacaranda.
Jalap., Jalapa.
Jatr., Jatropha.
Jug-c., Juglans cinerea.
Jug-r., Juglans regia.
Junc., Juncus.
Juni., Juniperus Virginiana.

K-ac., Kali aceticum.
K-ar., Kali arsenicosum.
K-bi., Kali bichromicum.
K-bro., Kali bromatum.
K-ca., Kali carbonicum.
K-chr., Kali chromicum.
K-clc., Kali chloricum.
K-cy., Kali cyanatum.
K-iod., Kali iodatum.
K-ma., Kali hypermanganicum.
Kali nitricum. See Nitrum.
K-sul., Kali sulphuricum.
Kalm., Kalmia.
Kiss., Kissingen.
Kou., Kousso.

Lab., Laburnum.
Lac-can., Lac caninum.
Lac-dfl., Lac defloratum.
Lach., Lachesis.
Lachn., Lachnanthes.
Lac-ac., Lactic acid.
Lact., Lactuca.
Lam., Lamium.
Laur., Laurocerasus.
Led., Ledum.
Lepi., Lepidium.
Lept., Leptandra.
Lil-t., Lilium tigrinum.
Linu., Linum.
Lip., Lipspringe.
Lith., Lithium.
Lith-m., Lithium muriaticum.
Lob-c., Lobelia cardinalis.
Lobel., Lobelia inflata.
Lob-s., Lobelia syphilitica.
Lon., Lonicera.
Lup., Lupulus.
Lyc., Lycopodium.
Lycpr., Lycopersicum.
Lycps., Lycopus.

Mac., Macrotinum.
Mag-c., Magnesia carbonica.
Mag-m., Magnesia muriatica.
Mag-s., Magnesia sulphurica.
Manc., Mancinella.
Mand., Mandragora.
Mang., Manganum.
Mang-m., Manganum muriaticum.
Mang-s., Manganum sulphuricum.
Marum, Marum verum.
Mec., Meconinum.
Medor., Medorrhinum.
Mela., Melastoma.
Meli., Melilotus.
Meni., Menispermum.
Ment-pi., Mentha piperita.
Ment-pu., Mentha pulegium.
Meny., Menyanthes.
Meph., Mephitis.
Merc., Mercurius.
Merc-ac., Mercurius aceticus.
Merc-br., Mercurius bromatus.
Merc-c., Mercurius corrosivus.

Merc-cy., Mercurius cyanatus.
Merc-d., Mercurius dulcis.
Merc-i-fl., Mercurius iodatus flavus.
Merc-i-r., Mercurius iodatus ruber.
Merc-m., Mercurius methylenus.
Merc-n., Mercurius nitrosus.
Merc-p-a., Mercurius precipitatus albus.
Merc-p-r., Mercurius precipitatus ruber.
Merc-s., Mercurius sulphuricus.
Merl., Mercurialis.
Mezer., Mezereum.
Mill., Millefolium.
Mit., Mitchella.
Morph.. Morphinum.
Mosch., Moschus.
Murx., Murex.
Mur-ac.. Muriaticum acidum.
Myric., Myrica.
Myris., Myristica.

Nat-ar., Natrum arsenicatum.
Nat-br., Natrum bromatum.
Nat-c., Natrum carbonicum.
Nat-hy., Natrum hypochlorosum.
Nat-m., Natrum muriaticum.
Nat-n., Natrum nitricum.
Nat-p., Natrum phosphoricum.
Nat-s., Natrum sulphuricum.
Naja, Naja.
Nap., Naphtha.
Narcot, Narcotinum.
Nicc., Niccolum.
Nicot., Nicotinum.
Nit-d-s., Nitri dulcis spiritus.
Nit-ac., Nitricum acidum.
Nit-m ac., Nitro-muriatic acid.
Nit-ox.. Nitrogenium oxygenatum.
Nitr.. Nitrum.
Nuph., Nuphar luteum.
Nx-m., Nux moschata.
Nx-v., Nux vomica.
Nym., Nymphæa odorata.

Oci., Ocimum.
Œna., Œnanthe.
Olnd., Oleander.
Ol-an., Oleum animale.
Ol-jec., Oleum jecoris aselli.
Op., Opium.
Opun., Opuntia.
Osm., Osmium.
Ost.. Ostrya.
Oxal-ac., Oxalicum acidum.
Ozo., Ozonum.

Pæon., Pæonia.

Pall., Palladium.

Pan., Panacea.

Par., Paris quadrifolia.

Pau-p., Paullinia pinnata.

Pau-s., Paullinia sorbilis.

Plb., Plumbum.

Ped., Pediculus.

Pen., Penthorum.

Per., Persica.

Peti., Petiveria.

Petr., Petroleum.

Petros., Petroselinum.

Phal., Phallus.

Phas., Phaseolus.

Phel., Phellandrium.

Phos., Phosphorus.

Ph-ac. Phosphoricum acidum.

Phys., Physostigma.

Phyt. Phytolacca.

Pic ac., Picricum acidum.

Pil., Pilocarpinum.

Pimp., Pimpinella.

Pin-c., Pinus cupressus.

Pin-l., Pinus lambertiana.

Pin-s., Pinus silvestris.

Pip-m., Piper methysticum.

Pip-n., Piper nigrum.

Plan., Plantago.

Plat., Platinum.

Plat-m., Platinum muriaticum.

Plect., Plectranthus.

Plmbg., Plumbago littoralis.

Podo., Podophyllum.

Polyg., Polygonum.

Pop., Populus.

Poth., Pothos.

Pru-p., Prunus padus.

Prun., Prunus spinosa.

Psor., Psorinum.

Ptel. Ptelea trifoliata.

Puls., Pulsatilla.

Pul-n., Pulsatilla nuttallina.

Pyrth., Pyrethram.

Pyrus, Pyrus.

Qua., Quassia.

Ran-b., Ranunculus bulbosus.
Ran-g., Ranunculus glacialis.
Ran-r., Ranunculus repens.
Ran-sc.. Ranunculus sceleratus.
Raph., Raphanus.
Ratan., Ratanhia.
Rheum, Rheum.
Rhod., Rhododendron.
Rhus, Rhus toxicondendron.
Rhus-r., Rhus radicans.
Rhus-v., Rhus venenata.

Ric., Ricinus.
Rob., Robinia.
Ros., Rosmarinus.
Rumx., Rumex crispus.
Ruta, Ruta.

Sabad., Sabadilla.
Sabin., Sabina.
Sac-alb., Saccharum album.
Sal-ac., Salicylicum acidum.
Sal-n., Salix niger.
Sal-p., Salix purpurea.
Samb., Sambucus.
Sang., Sanguinaria.
Sant., Santoninum.
Sap., Saponinum.
Sarr., Sarracenia.
Sars., Sarsaparilla.
Scam., Scammonium.
Scor., Scorpio.
Scut., Scutellaria.
Secale, Secale cornutum.
Sed., Sedinha.
Selen., Selenium.
Senec., Senecio.
Seneg., Senega.
Senn., Senna.
Sep., Sepia.
Serp., Serpentaria.
Sil., Silicea.
Sin-a., Sinapis alba.
Sin-n., Sinapis nigra.
Solnm., Solaninum.
Sol-a., Solanum arrebinta.
Sol-m , Solanum mammosum.
Sol-n., Solanum nigrum.
Sol-o., Solanum oleraceum.
Sol-p., Solanum pseudo-capsicum.
Sol-t., Solanum tuberosum.
Sol-t-æ., Solanum tuberosum ægrotans.
Spig., Spigelia.
Spig-m., Spigelia marilandica.
Spigg., Spiggurus.
Spira., Spiranthes.
Spire., Spirea ulmaria.
Spong., Spongia.
Squil., Squilla.
Stach., Stachys betonica.
Stann., Stannum.
Staph., Staphisagria.
Stict., Sticta pulmonaria.
Still., Stillingia sylvatica.
Stram., Stramonium.
Stront., Strontium.
Stry., Strychninum.
Sulph., Sulphur.
Sul-i., Sulphur iodatum.
Sul-ac., Sulphuricum acidum.
Sumb., Sumbul.
Syph., Syphilinum.

Tabac., Tabacum.
Tanac., Tanacetum.
Tang., Tanghinia.
Tann., Tannin.
Tarax., Taraxacum.
Tarent., Tarentula.
Tart-ac., Tartaric acid.
Taxus, Taxus baccata.
Tell., Tellurium.
Tep., Teplitz.
Tereb., Terebinthina.
Tet., Tetradymite.
Thal., Thallium.
Thea, Thea.
Ther., Theridion.
Thu., Thuja.
Tilia, Tilia.
Ton., Tongo.
Trif-p., Trifolium pratense.
Trif-r., Trifolium repens.
Trill., Trillium cernuum.
Trim., Trimethylaminum.
Trio., Triosteum.
Trom., Trombidium muscæ domesticæ.
Tus-f., Tussilago fragrans.
Tus-p., Tussilago petasites.

Upa., Upas.
Uran., Uranium nitricum.
Urea, Urea.
Urt-c., Urtica crenulata.
Urt-g., Urtica gigas.
Urt-u., Urtica urens.
Ustil., Ustilago.
Uva, Uva ursi.

Vac., Vaccininum.
Valer., Valeriana.
Verat., Veratrum album.
Verat-v., Veratrum viride.
Veratn. Veratrinum.
Verb., Verbascum.
Vesp., Vespa.
Vich., Vichy.
Vinc., Vinca.
Viol-od., Viola odorata.
Viol-tr., Viola tricolor.
Vip., Vipera.
Vip-l-f., Vipera lachesis fel.
Visc., Viscum album.

Wies., Wiesbaden.

Wild., Wildbad.
Wye., Wyethia.

Xanth., Xanthoxylum.

Yuc., Yucca.

Zing., Zingiber.

Ziz., Zizia.
Znc., Zincum.
Znc-a., Zincum aceticum.
Znc-c., Zincum cyanatum.
Znc-f., Zincum ferrocyanatum.
Znc-m., Zincum muriaticum.
Znc-p., Zincum phosphoratum.
Znc-s., Zincum sulphuricum.

